# No-Reflow after PPCI—A Predictor of Short-Term Outcomes in STEMI Patients

**DOI:** 10.3390/jcm9092956

**Published:** 2020-09-12

**Authors:** Larisa Renata Pantea-Roșan, Vlad Alin Pantea, Simona Bungau, Delia Mirela Tit, Tapan Behl, Cosmin Mihai Vesa, Cristiana Bustea, Radu Dumitru Moleriu, Marius Rus, Mircea Ioachim Popescu, Vladiana Turi, Camelia Cristina Diaconu

**Affiliations:** 1 Department of Medical Disciplines, Faculty of Medicine and Pharmacy, University of Oradea, 410073 Oradea, Romania; larisa.rosan@yahoo.com (L.R.P.-R.); rusmariusr@yahoo.com (M.R.); procardia_oradea@yahoo.com (M.I.P.); 2 Clinical County Emergency Hospital of Oradea, 410169 Oradea, Romania; v_cosmin_15@yahoo.com (C.M.V.); cristianabustea@yahoo.com (C.B.); 3 Department of Morphological Disciplines, Faculty of Medicine and Pharmacy, University of Oradea, 410073 Oradea, Romania; panteavladalin@yahoo.ro; 4 Department of Pharmacy, Faculty of Medicine and Pharmacy, University of Oradea, 410028 Oradea, Romania; 5 Department of Pharmacology, Chitkara College of Pharmacy, Chitkara University, Punjab 140401, India; tapanbehl31@gmail.com; 6 Department of Preclinical Disciplines, Faculty of Medicine and Pharmacy, University of Oradea, 410073 Oradea, Romania; 7 Department of Mathematics, Faculty of Mathematics and Computer Science, West University of Timisoara, 300223 Timisoara, Romania; radu.moleriu@e-uvt.ro; 8 Department of Cardiology, Faculty of Medicine, “Victor Babes” University of Medicine and Pharmacy, 30041 Timisoara, Romania; turi.vladiana@umft.ro; 9 Department 5, “Carol Davila” University of Medicine and Pharmacy, 050474 Bucharest, Romania; drcameliadiaconu@gmail.com; 10 Internal Medicine Department, Clinical Emergency Hospital of Bucharest, 105402 Bucharest, Romania

**Keywords:** acute myocardial infarction (MI), no-reflow phenomenon (NRP), primary percutaneous coronary intervention (PPCI), thrombolysis in myocardial infarction (TIMI) risk score

## Abstract

The no-reflow phenomenon following primary percutaneous coronary intervention (PPCI) in acute ST-elevation myocardial infarction (STEMI) patients is a predictor of unfavorable prognosis. Patients with no-reflow have many complications during admission, and it is considered a marker of short-term mortality. The current research emphasizes the circumstances of the incidence and complications of the no-reflow phenomenon in STEMI patients, including in-hospital mortality. In this case-control study, conducted over two and a half years, there were enrolled 656 patients diagnosed with STEMI and reperfused through PPCI. Several patients (*n* = 96) developed an interventional type of no-reflow phenomenon. One third of the patients with a no-reflow phenomenon suffered complications during admission, and 14 succumbed. Regarding complications, the majority consisted of arrhythmias (21.68%) and cardiogenic shock (16.67%). The anterior localization of STEMI and the left anterior descending artery (LAD) as a culprit lesion were associated with the highest number of complications during hospitalization. At the same time, the time interval >12 h from the onset of the typical symptoms of myocardial infarction (MI) until revascularization, as well as multiple stents implantations during PPCI, correlated with an increased incidence of short-term complications. The no-reflow phenomenon in patients with STEMI was associated with an unfavorable short-term prognosis.

## 1. Introduction

The “no-reflow” phenomenon (NRP) represents the myocardial tissue hypoperfusion after relief of occlusion, despite having an opened and patent epicardial coronary artery. It is essential to understand the pathophysiology of this phenomenon, to prevent and treat it early and thus, to avoid permanent myocardial damage and poorer prognosis. The steps in addressing this pathology consist of knowing the preventive measures to avoid the consequences of a possible failure at this stage, as well as a good knowledge of PNR diagnostic methods and treatment possibilities.

Following an acute ST-elevation myocardial infarction (STEMI), it is essential to achieve the reperfusion of the culprit artery as soon as possible, with the resumption of optimal blood flow. Primary percutaneous intervention (PPCI) is the most indicated reperfusion option in patients with acute STEMI, having an approximately 95% success rate in opening the occluded coronary artery [1]. Compared to thrombolytic therapy, PPCI is more effective and decreases mortality [2].

In several STEMI cases, despite the immediate opening of the culprit artery, the resumed blood flow is insufficient, leading to the onset of the NRP phenomenon and the incriminated factors include prolonged myocardial ischemia, consecutive endothelial dysfunction in the coronary microcirculation, and failure of proper blood flow restoration at this level. It is not a benign or sporadic condition, occurring in more than 20% of the patients undergoing PPCI for STEMI and in less than 2% of elective procedures [3]. In some cases, it is due to the failure of percutaneous coronary intervention (PCI), which has significant consequences in both the short and long term [4].

The NRP occurs in less than a quarter of patients with STEMI [5]. The numbers vary from 2% to 44% for NRP in PPCI and between 7.4% and 30.3% for subsequent mortality rates [6,7,8]. It can be transient and reversible if there is a prompt intervention during the percutaneous myocardial revascularization procedure [9], or it can be permanent, with a dramatic influence on the patient’s prognosis, both in the short and long term [10,11]. NRP following PPCI leads to higher 30-day mortality if not properly managed (32% vs 2.8%, *p* < 0.0.001) [12] and patients with TIMI flow from 0 to 2 have worse outcomes, even without significant epicardial obstruction [13], leading to prolonged myocardial ischemia and a 10-fold higher risk of clinical complications [14].

Short-term complications of the NRP consist of hemodynamic disturbances, cardiogenic shock, myocardial rupture, and as a consequence of the extension of the myocardial necrosis area, malignant heart rhythm, conduction disorders and acute pulmonary edema, leading over time to left ventricle (LV) remodeling in patients with subsequent heart failure or other arrhythmias, followed by premature death [15]. Fatal arrhythmias are the primary complications of patients with acute MI who develop NRP consecutive to prolonged myocardial ischemia, having the most complex and detrimental malignant arrhythmias in the first hours following the STEMI and decreasing with time [13]. Cardiogenic shock is the consequence of the extended myocardial necrosis and secondary rupture of the free myocardial wall or interventricular septum. At the same time, acute heart failure emerges as a result of the LV dysfunction, which correlates to the size of myocardial necrosis [16]. Patients who develop NRP have a higher death incidence compared to those without it, and those with a form of transient NRP also exhibit an increased risk of mortality, but only in the short term, whereas subjects with persistent NRP have an enhanced risk even in the long term [17]. Thus, NRP is an independent predictor of adverse clinical outcomes following acute MI, regardless of infarct size, and it is associated with heart failure and enhanced mortality.

The objectives of this research consisted of emphasizing the circumstances of the NRP in STEMI patients, regarding prevention, short-term outcomes and prognosis. We approached this topic given the urgent need for a better understanding of the situation (both in the laboratory and from a clinical point of view), but also because the limitations of the Romanian public health system (insufficient modern equipment in hospitals) in terms of patients’ evaluation, leading to the observation of specific aspects in these circumstances.

## 2. Materials and Methods

### 2.1. Study Design

Between January 2016 and March 2018, 942 patients with ST-elevation myocardial infarction (STEMI), presenting within 24 h from the symptom’s occurrence, were admitted in the Cardiology Clinic of the Clinical County Emergency Hospital of Oradea (Oradea, Bihor County, Romania).

The inclusion criteria were as follows: patients >18 year-old who agreed to participate in the research and signed the informed consent in this regard, with a STEMI diagnosis at the time of admission in the Cardiology Clinic of the Clinical County Emergency Hospital of Oradea, (Oradea, Bihor County, Romania), and who benefited from myocardial reperfusion through PPCI. The exclusion criteria consisted of mechanical failure to open the occluded coronary arteries, non-STEMI acute coronary syndromes, STEMI without PPCI (undergoing thrombolysis or coronary artery bypass surgery), pulmonary embolism, aortic dissection, acute cerebrovascular disease, refusal to participate in the study or to sign the informed consent, oncological or psychiatric disorders, patients <18 years or >90 years of age, and incomplete data.

**Figure 1:**
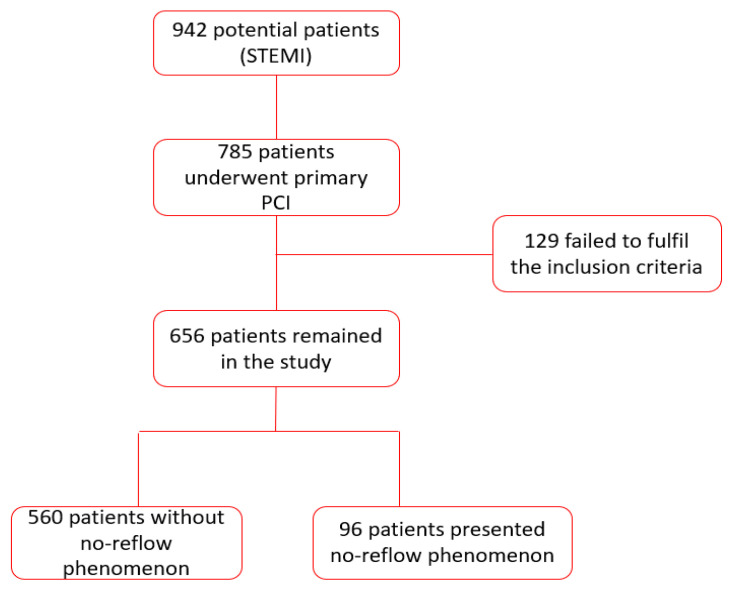
Flow chart describing the way of patient selection (STEMI: ST-segment elevation myocardial infarction, PCI: percutaneous coronary intervention).

After all the criteria were met, this research included 656 STEMI patients who underwent primary PPCI. The PCI patients were divided according to the presence/absence of the no-reflow phenomenon during the angiography procedure into NPR+ (*n =* 96) and NRP− (*n =* 560) groups. Figure 1 contains the flow chart presenting the patients’ selection.

The study included patients with NRP, both permanent and transient forms. We considered as an NRP patient any subject with STEMI and TIMI flow degree ≤2 and a degree of MBG ≤2 during angiography procedure. If the TIMI flow and MBG improved after administering vasodilators, we considered it transient NRP or, if not, as permanent NRP. Assessing NRP only through angiography underestimates the situation and the incidence because there is the myocardial form of the NRP, where patients have an excellent TIMI flow and MBG, but with insufficient myocardial perfusion. Other ways to detect NRP consist of ECG evaluation (if there is a persistent ST elevation >50–70% of the initial segment), myocardial contrast echocardiography, and cardiac MRI, etc. It is preferable to combine at least two modalities to assess NRP. Patients were followed throughout the hospitalization period, and ECGs were performed daily.

### 2.2. Ethical Statement

The research was carried out with the agreement of the Ethics Commission of the Oradea County Emergency Clinical Hospital of Oradea (Oradea, Bihor County, Romania), no. 1385/17.01.2019 and according to the principles of the Declaration of Helsinki [18].

### 2.3. Clinical Investigations

For the current study, we applied the international definition for STEMI diagnosis [19]. The most important criterion is the increased high-sensitivity cardiac troponin with at least one value over the 99th reference percentile of the upper limit. If this biomarker was modified, then it needed to search for another criterion, such as typical chest pain, electrocardiogram (ECG) modifications, altered echocardiographic parameters, or the presence of thrombus. ECG changes include new or presumably new ST-T wave changes, left bundle branch block or pathological Q waves. Echocardiography can show wall motion abnormalities and new or presumably new loss of viable myocardium. The thrombus can be directly visualized through angiography. The NRP in STEMI patients following PPCI is detected during the angiography procedure if thrombolysis in myocardial infarction (TIMI) score is ≤2 and the myocardial blush grade (MBG) is lower than 2, despite opening the culprit artery and placing the stent. The degree of TIMI flow is rendered subjectively, usually by the cardiologist who performs the intervention, by assessing the contrast substance passing through the investigated coronary artery. TIMI 3 is considered an optimal degree of flow, and it means the substance passes rapidly through the vessel, and the antegrade flow is identical before and after the barrier [20].

Unfortunately, TIMI flow assessment is not enough to evaluate and diagnose a NRP phenomenon. TIMI flow has a limited capacity to trace blood flow only in the epicardial arteries, not in the small arteries, and NRP happens mainly in the microcirculation. Many patients with good TIMI flow have NRP [21]; thus, another investigation is necessary. MBG is an imaging technique performed by an interventional cardiologist, who can assess during angiography the myocardial microcirculation and tissue reflow [22]. It tracks the opacification of the myocardial area with a contrast substance in the territory of MI. Thus, the contrast substance that quickly reaches the coronary microcirculation, achieving a “blush” appearance, signifies a good resumption of the blood flow in the microcirculation and equals a MBG 3 [16,23].

Applying a specific technique of myocardial revascularization depends on the interventional cardiologist who performs the procedure, and so does the interpretation of TIMI flow and the MBG. According to the European Society of Cardiology criteria, malignant heart rhythm disorders include ventricular tachycardia, paroxysmal ventricular tachycardia, and ventricular fibrillation [24]. Patients with cardiogenic shock develop tachycardia, dyspnea, tachypnea, and signs of hypoperfusion such as low blood pressure and low urine output [25]. Acute pulmonary edema is suspected based on symptoms and signs and is confirmed during a chest X-ray, showing typical pulmonary stasis. Left ventricle aneurysm may be found during echocardiography, showing wall segments with paradoxical systolic expansion [19].

The equipment and techniques used to perform the study followed the internal protocols of the clinic. Devices benefited from regular calibrations and check-ups. For ECGs, we used a BTL-08 MT Plus electrocardiograph (BTL, München, Germany) with 12 standard leads and added extra leads (right and posterior) if necessary, in selected cases. During admission, daily ECGs were performed or even multiple times/day if needed. Moreover, the percutaneous coronary intervention was performed in the cardiac catheterization laboratories, strictly following the procedures and standards, in sterile conditions and using the Philips Allura FD 10 angiograph (Philips, Cambridge, MA, USA). Drug-eluting stents were used for the patients included in the current study and did not employ any distal filter devices to protect from distal embolization. Based on the guideline recommendations [19], patients received dual antiplatelet therapy before PCI, either in the ambulance, emergency unit, or at the beginning of the admission. They received 300 mg of acetylsalicylic acid (BAYER Bitterfeld GmbH, Bitterfeld-Wolfen, Germany) and 300 mg Clopidogrel (EGIS Pharmaceuticals PLC, Budapest, Hungary) or 180 mg Ticagrelor (AstraZeneca, Södertälje, Sweden). During the procedure, if the TIMI flow was ≤2 and a MBG ≤ 2, resulting in an NRP phenomenon, the cardiologists attempted blood flow optimization. They injected eptifibatide into the coronary arteries (Agila Specialties Polska, Warsaw, Poland) or nitroglycerine (ZENTIVA, Hlohovec, Slovak Republic). Heart imaging consisted of echocardiography performed on a Siemens Acuson X300 device (Siemens Healthcare GmbH, Rudolstadt, Germany), before and after PCI; subsequently, follow-up echocardiography was performed 3–5 days after PCI.

Myocardial necrosis enzymes, which are mandatory for STEMI diagnosis, were quantified using the Architect ci4100 analyzer (Abbott, Palatine, IL, USA) for high-sensitive troponin I and determined through the spectrophotometric method. The Pathfast Compact Immuno-Analyzer (Mitsubishi Chemical Europe GmbH, Düsseldorf, Germany) was utilized for measuring troponin I from whole blood.

### 2.4. Statistical Analysis

At the beginning of this research, we described the data using contingency tables and column charts. The Kolmogorov–Smirnov test was used to evaluate the data distribution, and it revealed that the results were not normally distributed (*p* > 0.05) and so, further on, the non-parametrical test was used for this study. To compare two proportions, we applied the Z Test for Proportions. To compare the proportion obtained between the groups in this case-control study, the chi-square test was used, and the possible risk factors were calculated. At the end of this analysis, several regression models were tested; the exponential regression model was chosen (being the most appropriate) and applied, to find if there was an association between the possible complications presented in this study and the mortality rate. The power of the association was obtained from the Spearman rank-order correlation coefficient. For the entire study, the significance level was set at α = 0.05. The statistical analysis was performed using the SPSS v19 program.

## 3. Results

Both groups were homogenous in terms of age and gender, the majority being age between 51 and 70 years old and a smaller number younger than 50-year-old. The male gender was predominant. In the NRP+ group, the rate of high blood pressure, diabetes mellitus, dyslipidemia, and smoking were higher than in the control group. Prior Mis were a little more frequent in the NRP- patients than in the NRP+ patients. At discharge, the echocardiographic and ECG parameters were overall better in the NRP- group. During angiography, more than half of the patients with or without NRP (66.6%) had LAD as a culprit artery. In both groups, most patients had only the culprit vessel stented. In the NRP+ group, more than half received just one stent (54.16%), whereas in the NRP- group, most of the patients got two stents. The TIMI flow and MBG at the end of the procedure were lower in the NRP+ subjects. Most subjects in the NRP+ group received GPIIb/IIIa inhibitors and none in the control group. More patients from the NRP- group were treated in the first twelve hours compared to NRP+, and the rate of complications during hospitalization was higher for the latter. Of a total of 96 patients with NRP, 36 presented persistent coronary NRP and 27 the transient form. In comparison, 30 patients presented with the phenomenon of myocardial no-reflow.

All patients evaluated one hour after performing primary PCI showed a persistence between 50–70% of ST-segment elevation on the EKG pathway. It was found that patients with the transient form of no-reflow phenomenon showed a resolution of the ST segment on the ECG during follow-up. In contrast, in patients with a persistent coronary and myocardial type of no-reflow phenomenon, the persistence of ST-segment elevation between 50–70% of the initial value remained, with only small variations of the ST segment, with a resolution <50% of the initial ST-segment elevation throughout the hospitalization (Table 1).

Most malignant rhythm disorders developed either in the first 12 h after symptom onset, or after 24 h following PCI. Only a few patients had malignant arrhythmias in the 12–24 h-time interval. In addition to arrhythmias, other complications occurred. The most frequent was the cardiogenic shock, occurring in a significant number of patients with the NRP phenomenon. Less frequent complications were acute pulmonary edema, left ventricle aneurysm, responsive cardio-respiratory arrest or heart rupture. In the group of patients with NRP-, 15.53% developed complications during hospitalization, whereas in the NRP+ group, one third developed them. The culprit artery or the infarction localization were not considered factors that influenced the occurrence of complications (*p* > 0.05). The most encountered culprit artery in patients who developed complications during hospitalization was LAD for both groups. Most of them suffered rhythm and conduction disorders, and the others developed acute pulmonary edema, cardiogenic shock, early stent thrombosis and upper gastrointestinal bleeding, probably caused by the administration of dual antiplatelet therapy. The death incidence among those with NRP- equaled 25 cases, with a death rate of 4.46%, lower than the one in the NRP+ group, where the rate was 14.58% (Table 2).

**Figure 2:**
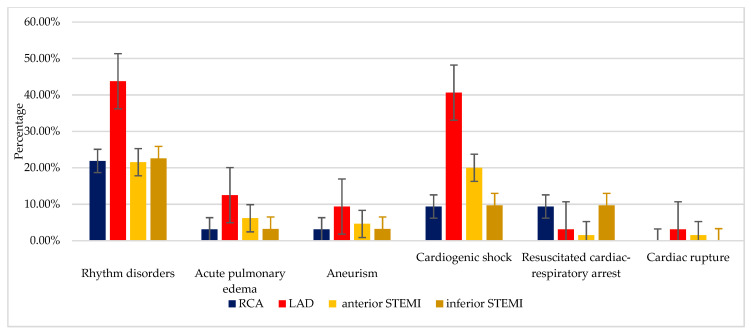
The prevalence of complications depending on the culprit artery and STEMI for NRP+ group Legend: RCA: right coronary artery (*n* = 27); LAD: left anterior descending artery (*n* = 64); STEMI: ST-segment elevation myocardial infarction (anterior STEMI: *n* = 65 and inferior STEMI: *n* = 31).

In this research, the left anterior descending artery was the main coronary artery responsible for the most in-hospital complications in patients with STEMI (Figure 2).

After the onset of typical STEMI symptoms and prompt PCI, most malignant arrhythmias started in <12 h from the first typical symptoms, and they were followed by cardiogenic shock as a secondary complication. After >12 h from the onset of STEMI, among the most encountered short-term complications were also malignant heart rhythm disorders. The percentage of complications in STEMI patients was significantly higher in the ones who had a longer time interval from the onset of the symptoms until reperfusion (>12 h). Depending on the number of implanted stents during PCI, there is a significant increase in the rate of short-term complications of patients who required the implantation of >2 DES (Table 3).

In this research, we also aimed to determine if the data were statistically significant, using the chi-square; a risk analysis was performed and the odds ratio (OR) parameters were calculated (the 95% confidence level was computed). In the first scenario, where the longer time-to-balloon was considered as a risk factor (RF) >12 h, a significant risk factor resulted for developing resuscitated cardiac-respiratory arrest. In the second scenario, where the number of implanted stents was considered a RF (*n* > 2), a significant risk factor was obtained for the rhythm disorders onset and for acute pulmonary edema. All the data are presented in Table 4.

**Figure 3:**
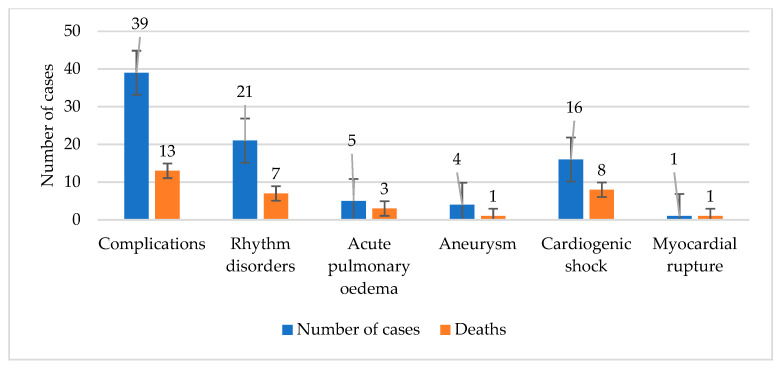
The dependence between the number of complications and the number of dead patients in the NRP+ group.

**Figure 4:**
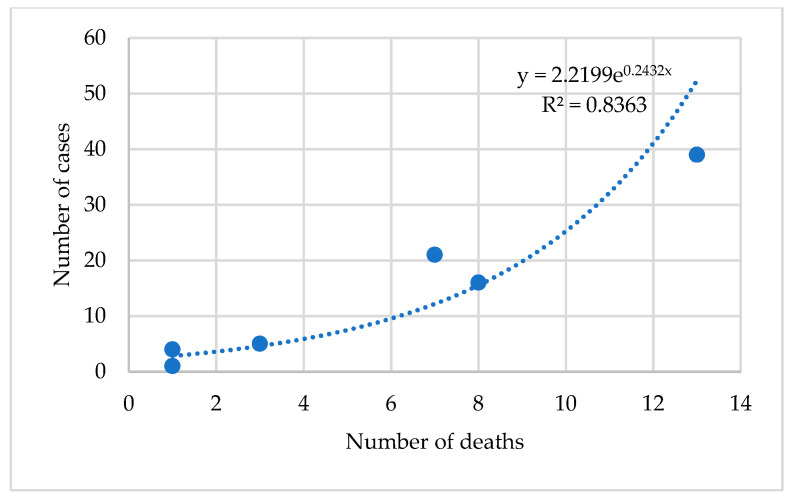
The association between the number of complications and the number of dead patients in the NRP+ group.

The possible association between complications and mortality rate was tested using the exponential regression model. The power of the correlation was given by the Spearman coefficient. A positive, strong, and extremely significant correlation (r = 0.915, R^2^ =0.813, *p* < 0.001) was obtained. All the results are presented in Table 5 and plotted in Figure 3 and Figure 4.

Regarding in-hospital mortality of patients with STEMI and NRP following PPCI, we found that one-third of patients developed complications and 14 of them succumbed, most frequently because of combined short-term complications.

## 4. Discussion

The post-PCI NRP in patients with STEMI predisposes to unfavorable prognosis both in the short and long term, with a significant decrease in life expectancy in these patients [17,26]. In the short term, the main complications consist of malignant heart rhythm disorders and cardiogenic shock, both leading to increased mortality in these patients. From the research of Ndrepepa et al. [27], it results that the NRP significantly contributes to the increase of in-hospital mortality, as well as long-term mortality.

The NRP incidence in this study was 14.6% in patients with STEMI and reperfusion by PPCI, out of which one-third developed in-hospital complications, and 1.4% died during admission. These results are consistent with the ones from other studies, which mention a low incidence of the NRP among patients with STEMI [5]. Moreover, the incidence of NRP is underestimated, mainly due to the insufficient use of modern diagnostic methods. It increases with the number of associated predisposing factors for the NRP. Thus, male patients, with arterial hypertension, diabetes mellitus, obesity, and smokers, have a higher risk of developing the phenomenon compared with those without these RFs. Moreover, the anterior STEMI with the left anterior descending artery as a culprit artery is a predisposing factor for NRP, according to previous research [6,26,28,29] and numerous existing studies that showed a direct correlation between the infarction size and NRP [30].

Although all patients enrolled in the study received dual antiplatelet therapy in the pre-hospitalization stage as the current guidelines recommend, the incidence of NRP remained high among patients with STEMI. Cenko et al. [31] concluded that the administration of antiplatelet therapy would significantly reduce the incidence of the NRP and also influences the mortality in these patients and the rest of pre-procedural medications (heparin, beta-blocker, and high dose statin), in order to improve the microvascular integrity of the coronary bed [32].

Regardless of the type of NRP, persistent (a degree of TIMI flow and MBG ≤2 post-PCI) or transient (present only during the PCI procedure, but resuming by the end of the procedure with a TIMI flow and MBG of more than 3), it significantly contributes to the occurrence of complications and unfavorable prognosis of these patients [27].

The post-procedural occurrence of hemodynamic instability in patients with NRP, according to Choo et al., can lead to malignant heart rhythm disorders, cardiogenic shock, or left ventricle aneurysm, followed by myocardial rupture [33], due to increased area of myocardial necrosis [17,34].

In the current research, there was a higher prevalence of anterior MI and left anterior descending artery as a culprit artery, compared to the other localizations, which were the main contributors to the MI-related NRP complications during admission [35]. According to Gupt and Gupta [16], an essential factor for NRP development is the symptom onset-to-balloon time longer than 12 h.

The time interval between the diagnosis of STEMI and myocardial reperfusion should be as short as possible. Ideally, we should aim at less than 120 min and acceptably less than 12 h from the onset of typical MI symptoms until reperfusion [19,24]. Patients’ prognosis remains closely related to the time elapsed from the onset of typical symptoms to PPCI [36]. Numerous studies showed that the appearance of the NRP correlates to prolonged myocardial ischemia and subsequent extension of the necrosis area [3,37]. However, as observed in this study, a period less than 12 h after the onset of MI can still be associated with NRP, especially in patients with multiple risk factors, such as diabetes [38], high blood pressure [39], chronic kidney disease [40,41], dyslipidemia, obesity, or tobacco use [42]. Although many data from the literature state that a long time between the onset of MI until the percutaneous myocardial revascularization causes the occurrence of the NRP, Kelbaek [43] considers that an optimal time interval from the onset of STEMI to PPCI does not influence the occurrence of the phenomenon.

Implantation of stents is preferable when performing percutaneous myocardial revascularization because it reduces the risk of reinfarction and repeated procedures [23,44]. Drug-eluting stents have been proven to be superior to bare-metal stents (BMS), and therefore, are recommended for PPCI in patients with acute MI [45]. We observed that the implantation of more than two stents during PCI procedures leads to the occurrence of the NRP, probably because these patients have multiple significant stenotic lesions or a large stenotic lesion, which requires deploying more stents. They are also more prone to endothelial dysfunction and distal embolization in the coronary microcirculation [3,46]. Thus, the NRP can be correlated to the increased number of stents placed in the culprit artery.

**Figure 5:**
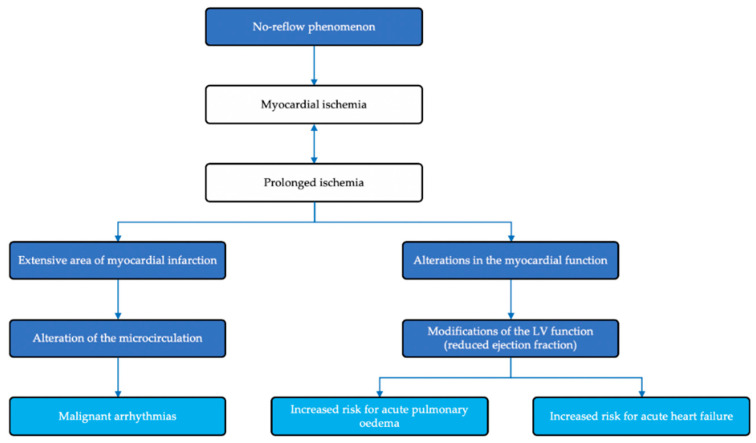
Pathophysiology mechanisms in the no-reflow phenomenon following primary percutaneous coronary intervention in ST-elevation myocardial infarction patients [47].

The appearance of malignant heart rhythm disorders in patients with NRP is the consequence of prolonged ischemia, with the occurrence of an area of extensive myocardial necrosis, which will further contribute to the alteration of coronary microcirculation. At the same time, the exposure of myocardial mass to prolonged ischemia will have as a consequence alterations of the contractile function, and subsequently of the left ventricle ejection fraction, increasing the risk of acute pulmonary edema and, in the long-term, heart failure. The pathophysiology mechanisms in the NRP phenomenon following PPCI in STEMI patients are summarized in Figure 5 [47].

Moreover, patients who develop NRP can exhibit a left ventricle remodeling process, mainly due to extensive areas of myocardial necrosis and secondary thinning of the wall, with the probability of developing a left ventricle aneurysm, which enhances, even more, the risk of mechanical complications, such as myocardial rupture.

The unfavorable outcomes occurred despite the repeated efforts of physicians to prevent the events. The cardiologists followed the protocols and performed all requested/necessary steps regarding the prevention and management of the NRP. As preventive measures, there was work on different fronts. First, there was a reduction of the door-to-balloon time as much as possible because no-reflow occurrence and its severity are time-dependent. Pharmacotherapy was another utilized tool. Dual antiplatelet therapy, intravenous 8000–10,000 U unfractionated heparin before/during PCI and statin in the maximum tolerated dose (80 mg atorvastatin) were administered. Another aspect consisted of controlling hemodynamic parameters (i.e., HR and BP) and other significant contributors (glycemia, electrolytes, and hydric balance) before, during, and after the PCI. If during PCI, TIMI flow was ≤2 and MBG was ≤2, cardiologists attempted blood flow optimization, through intracoronary eptifibatide or nitroglycerine administration. The motif for choosing eptifibatide in 68.75% cases was the evidence provided by results regarding better outcomes compared to conventional PCI and aspiration thrombectomy devices during primary PCI in STEMI patients, as is also highlighted in other published data [48]. Nitroglycerine was used in several cases, with unsatisfactory TIMI flow and MBG. This was similar to findings from previous studies, where nitroglycerin did not have a significant impact on the arteriolar tone and thus, on the no-reflow phenomenon. The reason for this outcome consists of the inability of metabolizing nitroglycerin and deriving nitric oxide at the microcirculation level, contrary to the epicardial arteries [49]. Vasodilators like verapamil, adenosine, or sodium nitroprusside, were not available in the hospital where this study was performed. Thus, the interventional cardiologist opted for intracoronary nitroglycerine and GP IIb/IIIa antagonists administered on a guide-catheter, but with unsuccessful results and the persistence of NRP. Thrombus aspiration was performed only in patients with a high thrombus burden.

In this study, the aim was to detect the NRP by evaluating the degree of TIMI flow and the MBG during angiography, following PPCI [50], as well as ST-segment elevation persistence of more than 50–70% despite a prompt reperfusion procedure [51]. The use of modern diagnostic modalities, including contrast echocardiography, myocardial magnetic resonance imaging, and radionuclide imaging techniques (such as SPECT—single-photon emission computed tomography and PET—positron emission tomography) can be of real use to correctly and accurately assess all patients who develop NRP [52,53].

Our research is consistent with data from the literature, but it shows that only a small proportion of patients with NRP succumbed during admission, possibly due to the modest size of the group of patients enrolled in the research. Kranenburg et al. [54] consider that the occurrence of the NRP in patients with STEMI contributes to subsequent severe myocardial dysfunction, and thus, to increased mortality at two years, while Morishima [13] considers it as a predictor of mortality at a mean follow-up of 5.8 years and Ndrepepa et al. of 5 years [28].

The strengths of the present study are in the identification and characterization of short-term complications during hospital admission in patients with STEMI. They develop NRP following PPCI, and thus, we emphasize the need for addressing these problems because most studies focus on the long-term complications. So far, this research is the first of its kind to address this topic in Romanian patients. Short-term complications (secondary to NRP) are often overlooked, although they are essential elements in the evolution of the disease.

It was observed that the no-reflow phenomenon did not occur when LCX was the culprit artery and thus, it highlights the importance in the post-PCI evolution of these patients but nonetheless, the small number of patients is a limitation of this finding and requires further research on larger groups. Another limitation is the lack of access to newer and more advanced diagnostic options, such as contrast echocardiography, myocardial scintigraphy, myocardial magnetic resonance imaging, and SPECT and PET techniques, which could have led to a higher prevalence of the NRP and a better quantification of the microvascular function.

The current research emphasizes the status of the patients from the north-west of Romania. Unfortunately, the information regarding the patients with STEMI and NRP is lacunar in this country; as far as we know, our research is the first in Romania related to STEMI patients, and very little research has been performed on this topic. According to the European Society of Cardiology, Romania is classified as a country at high risk of the development of cardiovascular disease and an increased death rate, with only five centers of interventional cardiology and a dedicated call 24/7 for the treatment of STEMI, for a population of about 19 million people. Thus, the number of cases with myocardial infarction that falls to each center is extremely high.

## 5. Conclusions

Following an acute MI with ST-segment elevation, prompt myocardial revascularization is paramount. The occurrence of NRP following PPCI leads to the unfavorable short-term prognosis by increasing the risk of complications and in some cases, of death. As a consequence, the NRP should not be underrated but considered as an essential marker of short-term outcomes, such as electrical or mechanical complications, and even death.

## Figures and Tables

**Table 1:** Clinical and angiographic features in patients with and without no-reflow phenomenon (NRP).

Clinical and Angiographic Features	All Patients		NRP+ Group		NRP− Group		*p* Value
	*n =* 656		*n =* 96		*n =* 560		
	No.	%	No.	%	No.	%	
**Clinical Features**							
Age (years)							
<50	199	30.33	18	18.75	181	32.32	* 0.008
51–70	278	42.38	43	44.79	235	41.96	0.603
>70	179	27.29	35	36.45	144	25.71	* 0.029
Male	432	65.85	64	66.6	368	65.71	0.857
HBP	448	68.29	70	72.91	378	67.5	0.294
Diabetes	187	28.5	42	43.75	145	25.89	* <0.001
Hyperlipidemia	418	63.71	82	85	336	60	* <0.001
Smoking (current)	289	44.05	56	58.33	233	41.6	* 0.002
Prior MI	27	4.11	3	3.12	24	4.28	0.596
**Echocardiographic Features**							
LVEF at discharge (%)							
lower EF ≤ 44	375	57.16	55	57.29	240	42.85	* 0.009
higher EF > 45	261	39.78	41	42.7	320	57.14	* 0.009
**ECG Features**							
ST resolution (%) at 1 h after PCI							
<50	560	85.36	0	0	560	85.36	* <0.001
>50–70	96	14.63	96	14.63	0	0	* <0.001
ST resolution (%) at discharge							
<50	69	10.51	69	71.87	0	0	* <0.0001
>50–70	587	89.48	27	28.12	560	100	* <0.0001
**Angiographic Features**							
Culprit artery							
LAD	351	53.5	64	66.6	287	51.25	* 0.005
RCA	229	34.9	27	28.1	202	36.07	0.131
LCX	76	11.58	5	5.2	71	12.67	0.035
Number of Affected Vessels							
One vessel	252	38.41	67	70	185	33	* <0.0001
Two vessels	286	43.59	24	25	262	46.78	* <0.0001
Three vessels	118	17.98	5	5	113	20.17	* <0.0001
Number of Stented Vessels							
One	543	82.77	87	90.62	456	81.42	* 0.028
Two	113	17.22	9	9.37	104	18.57	* 0.028
Three	0	0	0	0	0	0	-
Number of Stents							
One	195	29.72	52	54.16	143	25.53	* <0.0001
Two	363	55.33	35	36.45	328	58.57	* <0.0001
Three	98	14.93	9	9.37	89	15.89	0.097
Post PCI TIMI Flow							
0/1	37	5.64	37	38.54	0	0	* <0.0001
2	29	4.42	29	30.2	0	0	* <0.0001
3	590	89.93	30	3.12	560	100	* <0.0001
Post PCI MBG Flow							
0/1	30	4.57	30	31.25	0	0	* <0.0001
2	15	2.28	15	15.62	0	0	* <0.0001
3	611	93.14	51	53.12	560	100	* <0.0001
GPIIb/IIIa inhibitors	66	10.06	66	68.75	0	0	* <0.0001
**Time intervals**							
Symptom-to-device (min)							
≤720	561	85.51	71	73.95	490	87.5	* <0.0001
60–120	214	32.62	24	25	190	33.92	0.418
180–240	181	27.59	21	21.87	160	28.57	0.603
300–480	107	16.31	17	17.7	90	16.07	0.263
540–720	59	8.99	9	9.37	50	8.92	0.529
>720	95	14.48	25	26.04	70	12.5	* <0.0001
780–1440	64	9.75	11	11.45	53	9.46	* 0.004
>1440	31	4.72	14	14.58	17	3.03	* 0.004
STC	199	30.33	32	33.33	87	15.53	* <0.0001
**Culprit Artery of the Patients Who Developed STC**							
Culprit artery	119	18.14	32	4.87	87	15.53	
LAD	58	48.74	17	53.12	41	47.13	0.562
RCA	50	42.02	13	40.63	37	42.53	0.849
LCX	11	9.24	2	6.25	9	10.34	0.497

GP: glycoprotein; HBP: high blood pressure; LAD: left anterior descending artery; LCX artery: left circumflex; MI: myocardial infarction; NRP: no-reflow phenomenon; PCI: percutaneous coronary intervention; RCA: right coronary artery; STC: short-term complications; TIMI: thrombolysis in myocardial infarction; *p* values were obtained by applying the Z Test for Proportions; * *p* values < 0.05.

**Table 2:** Short-term complications in both groups.

Short-Term Complications	Group NRP+ *n =* 96		Group NRP− *n =* 560		*p* Value
	No.	%	No.	%	
Death	14	14.58	25	4.46	* <0.001
All short-term complications	32	33	87	15.53	* <0.001
Arrhythmias/conduction disorders	14	43.75	38	43.68	0.992
<12 h	7	50	21	55.26	0.728
13–24 h	2	14.29	4	10.53	0.704
>24 h	5	35.71	13	34.21	0.921
Acute pulmonary edema	5	15.63	17	19.54	0.624
Aneurysm	4	12.5	11	12.64	0.984
Cardiogenic shock	4	12.5	14	16.09	0.631
Resuscitated cardiorespiratory arrest	4	12.5	2	2.3	* 0.024
Myocardial rupture	1	3.12	0	0	0.969
Early stent thrombosis	0	0	3	3.45	0.289
Upper gastrointestinal bleeding	0	0	2	2.30	0.389

*p* values were obtained by applying the Z Test for Proportions; * *p* values < 0.05.

**Table 3:** Prevalence of complications depending on the time elapsed since the onset of STEMI and the number of implanted stents in NRP+ group.

Prevalence of Complications Depending on the Length of Time Elapsed Since the Onset of STEMI	≤12 h (*n =* 71)		>12 h (*n =* 25)		1 Stent (*n =* 52)		≥2 Stents (*n =* 44)	
	No.	%	No.	%	No.	%	No.	%
Heart rhythm disorders	14	19.72	7	28.00	7	13.46	14	31.82
Acute pulmonary edema	2	2.82	3	12.00	0	0.00	5	11.36
Aneurysm	2	2.82	2	8.00	3	5.77	1	2.27
Cardiogenic shock	9	12.68	7	28.00	7	13.46	9	20.45
Resuscitated cardiac-respiratory arrest	0	0	4	16.00	2	3.85	2	4.55
Cardiac rupture	0	0	1	4.00	1	1.92	0	0.00

STEMI: ST-segment elevation myocardial infarction.

**Table 4:** The risk factors in two different scenarios (1st scenario—time as a risk factor; 2nd scenario—the number of stents as a risk factor), for all the tested complications in the NRP+ group.

Prevalence of Complications Depending on the Artery Involved in STEMI	Risk Factor (The Risk Exposure)			
	Time (>12 h)		The Number of Stents (>1 Stent)	
	OR, 95%	*p* Value	OR, 95%	*p* Value
Heart rhythm disorders	OR=1.58, 95%∈(0.55;4.52)	0.4	OR=3, 95%∈(1.08;8.31)	* 0.04
Acute pulmonary edema	OR=4.71, 95%∈(0.74;29.99)	0.1	OR=6.67, 95%∈(1.03;59.38)	* 0.02
Aneurism	OR=3, 95%∈(0.39;22.52)	0.27	OR=0.37, 95%∈(0.03;3.78)	0.62
Cardiogenic shock	OR=2.68, 95%∈(0.87;8.19)	0.12	OR=1.65, 95%∈(0.56;4.88)	0.42
Resuscitated cardiac-respiratory arrest	OR=13.33, 95%∈(1.41;125.8).	* 0.02	OR=1.18, 95%∈(0.17;8.05)	0.99
Cardiac rupture	OR=2.92, 95%∈(0.17;48.46).	0.46	OR=1.18, 95%∈(0.08;18.35)	0.99

STEMI: ST-segment elevation myocardial infarction; * *p* < 0.05.

**Table 5:** The exponential regression model for the NRP+ group.

ANOVA Test	Sum of Squares	df	Mean Square	F Statistic	*p* Value
Regression	10.143	1	10.143	35.773	* <0.001
Residual	1.985	7	0.284		
Total	12.128	8			
The independent variable is “Deaths”.					

The strength of the association was computed by using the Spearman coefficient = 0.915 and R square = 0.813. For the statistical significance, the ANOVA test was applied; df: degree of freedom; * *p* values < 0.05.
